# Effect of Surgical Humidification on Inflammation and Peritoneal Trauma in Colorectal Cancer Surgery: A Randomized Controlled Trial

**DOI:** 10.1245/s10434-022-12057-3

**Published:** 2022-07-06

**Authors:** Shienny Sampurno, Timothy Chittleborough, Meara Dean, Michael Flood, Sandra Carpinteri, Sara Roth, Rosemary M. Millen, Helen Cain, Joseph C. H. Kong, John MacKay, Satish K. Warrier, Jacob McCormick, Jonathon G. Hiller, Alexander G. Heriot, Robert G. Ramsay, Andrew C. Lynch

**Affiliations:** 1 Sir Peter MacCallum Department of Oncology, The University of Melbourne, Victoria, Australia; 2 Peter MacCallum Cancer Centre, 305 Grattan Street, Melbourne, Victoria 3000 Australia; 3 Epworth Healthcare, Richmond Victoria, Richmond, Australia

## Abstract

**Background:**

Pre-clinical studies indicate that dry-cold-carbon-dioxide (DC-CO2) insufflation leads to more peritoneal damage, inflammation and hypothermia compared with humidified-warm-CO_2_ (HW-CO2). Peritoneum and core temperature in patients undergoing colorectal cancer (CRC) surgery were compared.

**Methods:**

Sixty-six patients were randomized into laparoscopic groups; those insufflated with DC-CO2 or HW-CO2. A separate group of nineteen patients undergoing laparotomy were randomised to conventional surgery or with the insertion of a device delivering HW-CO2. Temperatures were monitored and peritoneal biopsies and bloods were taken at the start of surgery, at 1 and 3 h. Further bloods were taken depending upon hospital length-of-stay (LOS). Peritoneal samples were subjected to scanning electron microscopy to evaluate mesothelial damage.

**Results:**

Laparoscopic cases experienced a temperature drop despite Bair-Hugger^TM^ use. HW-CO2 restored normothermia (≥ 36.5 °C) by 3 h, DC-CO2 did not. LOS was shorter for colon compared with rectal cancer cases and if insufflated with HW-CO2 compared with DC-CO2; 5.0 vs 7.2 days, colon and 11.6 vs 15.4 days rectum, respectively. Unexpectedly, one third of patients had pre-existing damage. Damage increased at 1 and 3 h to a greater extent in the DC-CO2 compared with the HW-CO2 laparoscopic cohort. C-reactive protein levels were higher in open than laparoscopic cases and lower in both matched HW-CO2 groups.

**Conclusions:**

This prospective RCT is in accord with animal studies while highlighting pre-existing damage in some patients. Peritoneal mesothelium protection, reduced inflammation and restoration of core-body temperature data suggest benefit with the use of HW-CO2 in patients undergoing CRC surgery.

**Supplementary Information:**

The online version contains supplementary material available at 10.1245/s10434-022-12057-3.

Effective laparoscopic and robot-assisted abdominal surgery depends upon consistent insufflation using carbon dioxide (CO_2_) delivered from a cylinder as a dry-cold gas (DC-CO2). Studies in animals and with human participants report a range of physiological, anatomical and immunological effects associated with the desiccating nature of DC-CO2. These are ameliorated by reduced pressure and/or the addition of humidification.^1–3^

Translating the effects of insufflation observed in pre-clinical studies to patients is problematic and some key clinical concerns cannot be reliably determined in animals; for instance, pain management and length of hospital stay (LOS). It would appear that reducing laparoscopic pressure alone affords multiple clinical and health-economic benefits as reported in a phase 3 RCT.^4^ Others have examined a range of parameters such as pain in patients undergoing colorectal surgeries, finding no benefit in terms of reduction with HW-CO2 compared with DC-CO2^5^ or some benefit more generally with abdominal surgery as reviewed by Binda.^6^ There is a modest benefit in maintaining normothermia with HW-CO2.^7^

HW-CO2 may reduce the inflammatory state in patients undergoing abdominal surgery as measured by interleukin 6 (IL6) induction.^2^ Another clinical parameter is an associated ~50% reduction of surgical site infections in patients undergoing laparoscopic surgery with HW-CO2 compared with DC-CO2.^8^ One robust metric to investigate differences between these two gas modalities is to evaluate peritoneal integrity during the operative experience, whereby the tissue state is measured at the ultra-structural level by scanning electron microscopy (SEM).^9,10^ Biopsies can be processed and scored by investigators blinded to the treatment to compare multiple experimental parameters across species. Two parameters are informative; mesothelial cell damage such as bulging or delamination away from the basement matrix, as well as microvillus integrity.^10–14^

An additional, but not proven, consideration is that peritoneal damage may predispose to the adherence of tumour cells to the peritoneal wall, thus seeding peritoneal carcinomatosis. Animal studies support this proposition with evidence that HW-CO2 reduces tumour cell embedding and tumour establishment.^10,11,15–18^ It is notable that tumour cells are evident at commencement of surgery in humans.^19^ However, cancer-associated benefit from HW-CO2 has not been confirmed in patients undergoing colorectal or other surgery.^20^ In this RCT patients operated upon for CRC were investigated by experienced CRC surgeons together with laboratory-based scientists involved in preclinical studies. Metrics indicate that HW-CO2 impacts systemic inflammation and peritoneal cell integrity while highlighting pre-existing peritoneal damage in a substantive proportion of patients.

## Methods

**Fig. 1:**
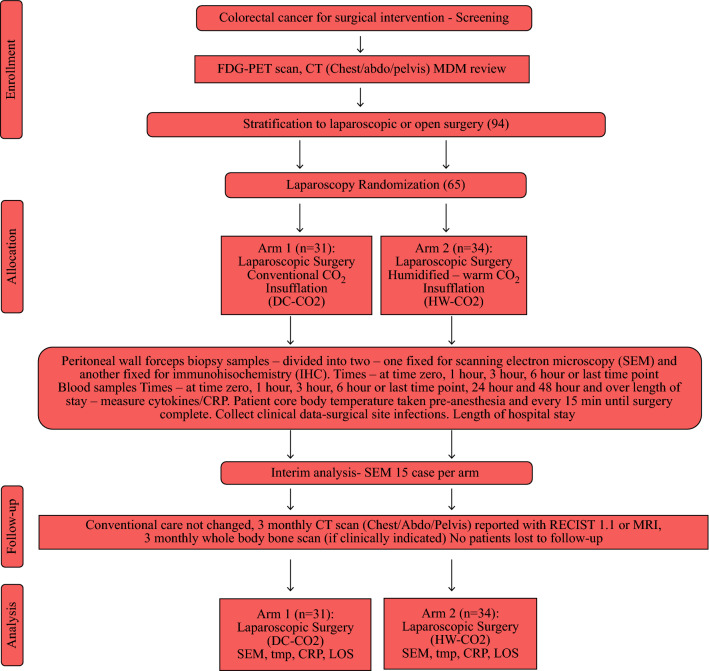
CONSORT flow chart describing laparoscopic arms

Patients enrolled were undergoing elective laparoscopic colonic or rectal resections for CRC at two hospitals; Epworth Healthcare, and the Richmond and Peter MacCallum Cancer Centre (PMCC) between 2016 and 2019. An additional smaller group was assigned to open surgery. Patients were included if 18 years or older with written informed consent. Exclusion criteria included patients under age 18, those with known intra-abdominal sepsis, a pre-operative steroid dependence, being pregnant, a prior diagnosis of Crohn’s disease or ulcerative colitis, an inability to consent due to a cognitive/language barrier, or having had a pre-operative blood transfusion. *Cohort 1:* 31 patients—laparoscopy with DC-CO2. *Cohort 2:* 34 patients—laparoscopy using HW-CO2; *Cohort 3:* 9 patients for surgery via laparotomy, no CO_2;_ *Cohort 4:* 10 patients undergoing surgery via laparotomy with delivery of HW-CO2 into the open abdominal cavity. An interim analysis of temperature and peritoneal samples was planned after recruitment of 15 patients in cohorts 1 and 2 (Fig. 1). Perioperative morbidity was evaluated using the Clavien-Dindo Classification.^21^ Exploratory studies on open cases are described in Supplementary Fig. S1. Patient characteristics are tabulated (Table 1).

**Table 1:** Procedures and patient characteristics

	HW Laparoscopic			DC Laparoscopic		*p values*	HW Open		Conventional open		*p values*
Patients^&^	34			31		–	9		10		–
Time of insufflation (min)	195 +/− 11			178 +/− 16		0.36	256 +/− 28		(291 +/− 27)		0.43
Volume of CO_2_ (l)	2111 +/− 165			1922 +/− 203		0.48	2190 +/− 259		N/A		
Robot-assisted	19			16		0.73	N/A		N/A		–
Cancer location											
Colon Ca	8 (24%)			14 (52%)		–	4 (50%)		4 (40%)		–
Rectum Ca	26 (76%)			15 (48%)		0.06	4 (50%)		6 (60%)		> 0.99
Sex	Male		Female	Male	Female		Male	Female	Male	Female	
Number	19		15	20	11	0.61	6	3	7	3	0.63
Age (mean)	64		60	67	66	0.60	68	54	64	57	0.52
BMI (mean)	27		29	26	27	0.52	25	22	30	32	0.14
Pre-existing peritoneal damage*	17%			19%		ns	22%		20%		ns
Surgical site infections	0			5		0.02	3		2		> 0.99
Anastomotic leak	0			1		0.47	0		4		0.05
Sepsis or SIRS (<30 days)	5			4		> 0.99	4		4		> 0.99
Length of stay (median)	8			8.5		*–*	15		17.5		
mean +/- SEM	10.0 +/− 1.0			10.9 +/− 1.3		0.28	21.4 +/− 6.6		22.7 +/− 4.4		0.44
Total amount of fluids mean +/- SEM	2333 +/− 136			2303 +/− 227		0.45	3318 +/− 618		4050 +/− 556		0.20
Severe pain 10-1 over 24 h post-op: 10 = none; 0 = all the time	2			1.6		0.24	2		1.3		0.18
median	1			1		–	1		1		–
ASA score (%)	0			0		>0.99	0		0		> 0.99
I	19			21		0.59	4		7		0.26
II	13			10		0.40	3		3		0.63
III	30			25		–	5		8		–
DFS (cases) 3-year DFS	82%			72%		0.93	nd		nd		–
Median follow-up	2.4 years			2.1 years		–	nd		nd		–
Tumour recurrence cases^#^	4			6		0.31	4		2		0.26

^***&***^Laparoscopic cases were randomised separately to open cases.

^*^Peritoneal SEM showing damage in both mesothelial cell bulging/delamination and microvillus damage or loss; *nd* - not determined

^#^Only one case had a peritoneal recurrence

^Δ^Duration of open conventional surgery

Patients underwent laparoscopic surgery with or without robotic assistance involving hemicolectomy for colon cancer and low or ultra-low anterior resection (ULAR) with or without loop ileostomy for rectal cancer. Insufflation pressure was at 12 mmHg. Laparotomy cases ranged from pelvic and posterior exenteration, ULAR to right hemicolectomy. HW-CO2 was delivered from the insufflator by a HumiGard^TM^ MR860 device (Fisher and Paykel Healthcare, Auckland New Zealand) or by a diffuser to the open abdomen in laparotomy cases immediately after the abdomen was opened. HumiGard^TM^ used in this study delivered at a minimum 33 mg/l dH_2_O per min at a minimum temperature of 35 °C.

Patients were anesthetized with a standardized balanced anaesthetic (inhaled 60/40: air/oxygen mixture) consisting of total intravenous anaesthesia with propofol-infusion typically titrated to an estimated plasma concentration of 4-5 mg/ml, intravenous fentanyl, oxycodone or morphine, and intravenous paracetamol 1000 mg at the anaesthetist’s discretion. Patients were offered a pre-operative injection of intrathecal morphine 150–200 mg (+/− intrathecal local anaesthesia) for post-operative analgesia. Local anaesthesia infiltration, intra-operative dexamethasone (4–8 mg), ketamine and non-steroidal anti-inflammatory agents were administered at the anaesthetist’s discretion. Patients had not received neoadjuvant chemotherapy and/or radiotherapy prior to surgery.

The central questions were to address the effect of HW-CO2 on core-body temperature, peritoneal damage and inflammation. For a comparison of proportions between arms in a two-arm trial, a sample size of 120 per arm was considered sufficient to detect a fairly small effect size of 0.36 with a power of 0.8 regarding peritoneal damage. If the overall proportion across both arms was in the vicinity of 0.5, this was enough to detect an approximate 18% difference in proportions between arms. These calculations were based on mouse data following 1 h of treatment; the maximum time permitted by the institutional animal ethics committee.^11^

After assessment by the multidisciplinary team members (MDM) patients were randomized for each hospital site and by surgical approach either laparoscopic or open. Patients were assigned to laparoscopic or open surgery by the MDM and then randomised. Randomisation was blocked to achieve even numbers in each group of HW-CO2 and DC-CO2 for laparoscopic cases or HW-CO2 perfusion or conventional laparotomy. Patients were randomize using www.random.org.

A planned interim analysis of the first 34 laparoscopic cases randomized to HW-CO2 and DC-CO2 was conducted, finding a modest but significant difference in damage measured at 3 h post initiation of surgery. Using these human data it was found that 21 cases in each arm would be sufficient to achieve 95% confidence and 80% power if 3-h cases were compared. To cover the unexpected level of pre-surgery peritoneal damage, the number of cases was increased above the theoretical number of patients undergoing laparoscopic surgery to 31 in the DC-CO2 arm and 34 in the HW-CO2 arm; as driven by the randomization protocol. The study was paused due to the COVID-19 pandemic and was not re-initiated based upon having reached sufficient cases to assess peritoneal damage.

Data were collected prospectively to include symptoms, age, weight and sex. The use of pharmaceutical agents for pain and inflammation management were recorded. Central data were held at PMCC or Epworth Healthcare and were provided to investigators after experimental data were generated, recorded and analysed. Race and ethnicity data were not collected as this was not customary or required in Australia at the time the RCT was approved.

*Primary objectives:* to determine whether insufflation of HW-CO2 results in reduced peritoneal tissue damage, as measured by percentage of normal microvilli remaining, degree of peritoneal mesothelial cell morphological change and existing peritoneal damage.

*Secondary objectives:* to test for a relationship between humidification and markers of systemic inflammation by measurement of plasma levels of C-reactive protein (CRP), circulating IL6, IL8, IL10 and TNFα. Intra-operative and post-operative core temperature were to be recorded whereby HW-CO2 was expected to maintain perioperative normothermia. HW-CO2 was predicted to reduce LOS.

All data are expressed as mean ± standard error of the mean evaluated using GraphPad Prism 9 and analysed by 1- or 2-way ANOVA with Tukey’s multiple comparisons test or one-tailed unpaired *t*-test or by chi-square analysis. A *p*-value of less than 0.05 was considered statistically significant. Survival was plotted as a Kaplan-Meier graph. The study was sponsored by Epworth Healthcare research, and human ethics approval was obtained from the Epworth (677-15) and Peter MacCallum Cancer Centre (LARF/52753/PMCC-2019) and registered retrospectively at Australian clinicaltrials.gov.au submission no. 382831.

Tissue specimens (5 × 5 × 1 mm) were taken from the peritoneal wall with forceps distant from the region of the operation and were processed as described.^11,22^ Damage was determined by viewing SEM micrographs. Two features were evaluated using an extent metric, and scored blinded-to-coding by two scientists independently: mesothelial cell-bulging or detachment/retraction, and microvillus damage-shortening and/or loss; 0 (0–5%), 1 (6–25%), 2 (26–50%), 3 (51–75%) or 4 (> 76%). Both scores (0–4) can stand alone or be added to produce a scale of 0–8. Cytokines/chemokines were analysed using the LEGENDplex^TM^ Human Inflammation Panel 1 kit (BioLegend, Catalog# 740809 USA) using the BD FACS Verse flow cytometer (BD Biosciences, USA) and analysed with LEGENDplex™ Data Analysis Software Suite Version 2021.07.01 (BioLegend, USA). C-reactive protein (CRP) levels were determined using the Multigent CRP Vario kit (Sentinel Diagnostics, Italy) and on the Abbott Architect c4000-clinical chemistry analyser (Abbott, USA) by the pathology laboratory at PMCC.

## Results

**Fig. 2.**
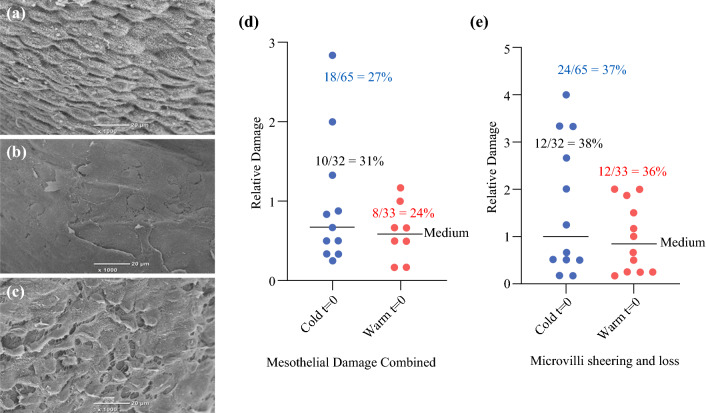
Scanning electron micrographs showing **a** normal peritoneal mesothelium, **b** loss of microvilli and **c** mesothelial cell retraction in pre-surgical samples. Relative damage to mesothelial cells **d** and loss or damage of microvilli **e**. Percentages of total samples with damage noted

**Fig. 3.**
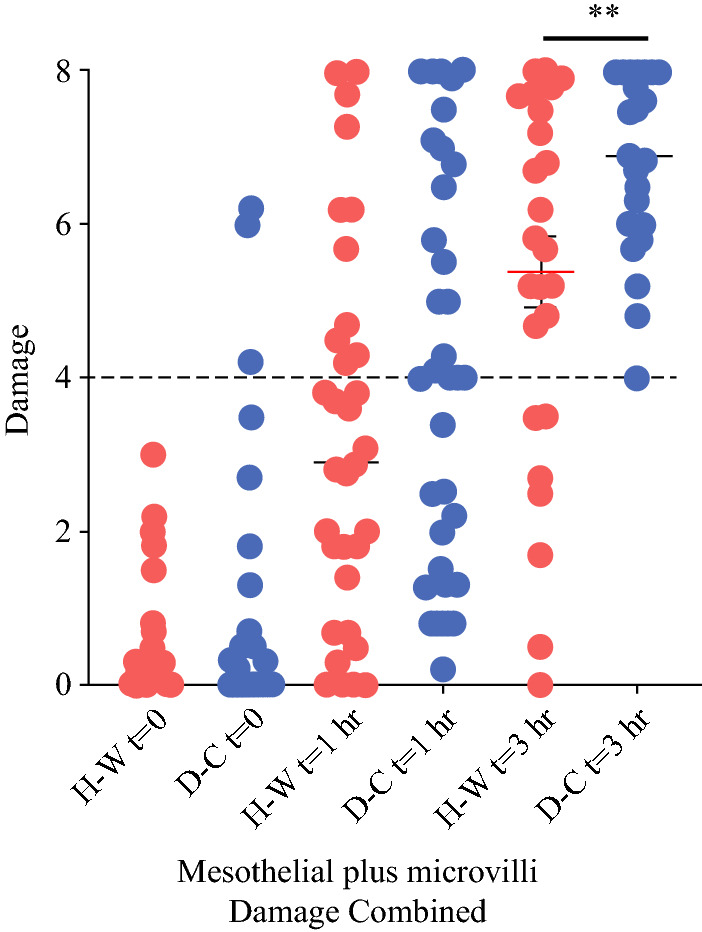
Combined peritoneal mesothelial and microvilli damage following insufflation with humidified-warm or dry-cold CO2 over 3 h. Mean +/− SEM shown for each time point. (**p = 0.0127 *t*-test, two-tailed)

An objective and quantifiable measure of peritoneal health and injury can be obtained by assessing the ultrastructure of the mesothelial layer by SEM.^11,12^ An interim analysis exploring the first 15 laparoscopic patients in the HW-CO2 and DC-CO2 groups was planned. Unexpectedly some baseline/pre-surgical samples showed damage that had not been a feature of previous animal studies. SEM micrographs depicting anticipated normal morphology (Fig. 2a) and damage in baseline samples are shown in Fig. 2b and c. Approximately 20% of samples had moderate to substantial pre-existing damage. The median damage was equivalent across groups (Fig. 2d and e). Samples were taken at 1 and 3 h. As expected, based upon preclinical studies, damage increased over time. When considered separately, mesothelial and microvillus damage were found to be significantly greater in the DC-CO2 group compared with the HW-CO2 group, respectively (*p* = 0.0056 and *p* = 0.0168; *t*-test one-tailed) at 3 h but not 1 h (Fig. S2A and B). The collective damage of the DC-CO2 group compared with the HW-CO2 group at 3 h was significant (**p = 0.0127 *t*-test two-tailed). On inspection of the distribution of cases with peritoneal damage at 3 h it was notable that seven cases in the HW-CO2 group were below 50% damage levels while none were found in the DC-CO2 group: perhaps indicative of some degree of peritoneal protection (Fig. 3).

About half of the laparoscopic cases were robot-assisted and when examined separately, damage was found to be significantly greater in the DC-CO2 group compared with the HW-CO2 group (*p* = 0.0279; *t*-test; one-tailed; data not shown). Although approximately 20% of cases had existing peritoneal damage this did not predispose for more extensive subsequent damage, nor where these cases were excluded did the effect of HW-CO2 on reduced accumulated damage compared with the DC-CO2 group substantially diminish. Although did not recruit the planned open cases assessment of pre-existing peritoneal damage and temperature data provided additional information.

Multiple drugs were employed to mitigate against inflammation and/or pain. The distribution of drug-treatment among patients is shown in Supplementary Fig. S3. No significant differences between patient groups were found, with scores mostly in the higher 2–3 range (0–10) indicative of effective, but required, severe pain control (Table 1).

**Fig. 4.**
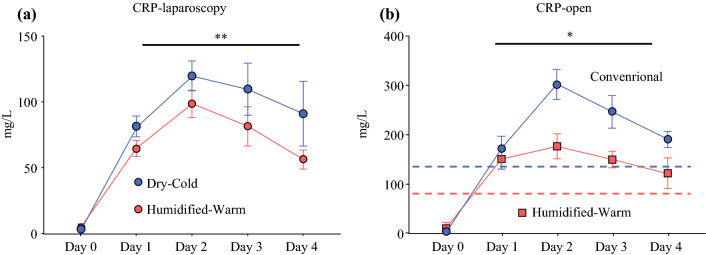
CRP levels in serum over 4 days post-surgery in **a** laparoscopic and **b** open surgery cases. Mean +/− SEM shown for each time point. (**p = 0.0041 and *p = 0.0001, respectively, paired t-test; ANOVA)

Baseline-CRP was within the normal low values range and rose by day 1, continuing for several days. As a group, the CRP levels post-surgery in the DC-CO2 group remained significantly higher for the first 4 days compared with the HW-CO2 groups (*p* = 0.0041: ANOVA). This was evident in the laparoscopic surgery groups and laparotomy cohorts (*p* = 0.0001; ANOVA) with the notable greater impact of open surgery on CRP levels, which on average were double that of the laparoscopic groups (Fig. 4a and b).

CRP production by the liver is mediated by interleukin 6 (IL6).^23^ The range of IL6 is 1–5 pg/ml in healthy individuals and was comparable to the pre-operative blood levels in most patients in this study, which increased sharply in patients undergoing laparoscopic surgery at 24 and 48 h. The difference between HW-CO2 (mean = 75 and 42 pg/ml; 24 and 48 h, respectively) and DC-CO2 (mean = 151 and 97 pg/ml; 24 and 48 h, respectively) was not statistically different (*p* = 0.087; one-sided *t*-test) **(**Supplementary Fig. S4). Patients undergoing open surgery on average had higher IL6 levels at 24 and 48 h compared with the laparoscopic cases; HW-CO2-open (mean = 133 pg/ml) and conventional open (mean = 319 pg/ml). This was not statistically significant. Additional cytokines/chemokines were evaluated pre-surgery (baseline) finding increases in IL8, IL10, MCP-1and IL18 in both laparoscopic cohorts, but no significant differences between groups were observed (Supplementary Fig. S5 and 6).

**Fig. 5.**
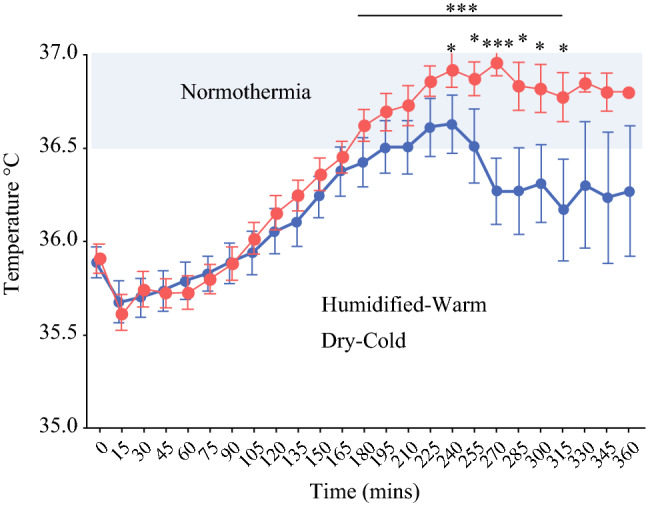
Patient core body temperature following insufflation showing normothermia range used by NICE guidelines UK. Mean +/− SEM shown. (***Paired analysis across times shown *p* = 0.0002 one-way ANOVA

All patients were warmed with a Bair Hugger^TM^ prior to surgery and monitored every 15 min until return to recovery. Starting temperatures ranged widely. When considered as groups subjected to DC-CO2 and HW-CO2 insufflation, on average patients remained below 36 °C for the first 90 min, with an initial temperature drop at 15 min. Seven DC-CO2 and two HW-CO2 remained in the hypothermic range (Supplementary Fig. S7A and B). Parallel temperature recovery was evident for both groups as they approached normothermia occurring at 180 min. However, the DC-CO2 group lost this trajectory and collectively fell below 36.5 °C for the remaining monitoring period. The HW-CO2 group showed significantly higher (*p* = 0.0002; one-way ANOVA) temperatures than the DC-CO2 group between 240 and 315 min; beyond these times insufficient cases were available for statistical analysis (Fig. 5). Open surgery case cohort sizes were quite small but revealed a trend where conventional open cases on average failed to reach 36.5 °C in the theatre and in this study the HW-CO2 group started at 0.4 °C colder (Supplementary Fig. S8). The relative core temperature increase appeared to be more rapid in the HW-CO2 group. Theatre temperature was between 20 and 22 °C.

**Fig. 6.**
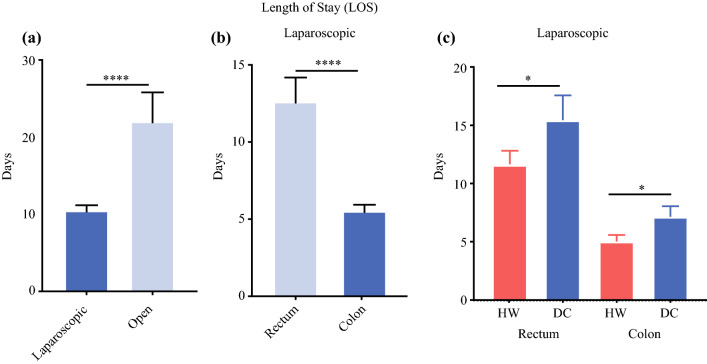
Length of hospital stay in **a** laparoscopic verses open cases, **b** those cases with cancers of the rectum verses colon and **c** those laparoscopic cases with cancers of the rectum or colon insufflated with different CO_2_ gas modalities. (**** *p* > 0.0001 two-tailed *t*-test; *p = 0.027-colon, *p* = 0.0474-rectum one-tailed *t*-test)

LOS in the laparoscopic group was on average 10.5 days compared with 22 days for the open cases (Fig. 6a) (*p* < 0.0001; *t*-test). Twenty-nine cases were robot-assisted and 37 were conventional laparoscopic procedures whereby LOS was not different (data not shown). When the cancer status of patients in the laparoscopic group were examined, 37 were colon and 27 rectal, and in view of the expected longer LOS in the rectal group this was confirmed, finding it to be 13.1 days in the rectal group versus 6.0 days for the colon group (Fig. 6b) (*p* < 0.0001; *t*-test). Accordingly, LOS was explored for rectal or colon laparoscopic surgery based upon type of insufflation gas. LOS in the HW-CO2 rectal group was 11.6 days compared with 15.4 days in the DC-CO2 rectal group (*p* = 0.027, *t*-test; one-tailed) and 5.0 days in the HW-CO2 colon group compared with 7.2 days in the DC-CO2 colon group (Fig. 6c) (*p* = 0.047, *t*-test; one-tailed). CRP levels were explored by tumour site as well as presence of sepsis and these did not explain the difference between HW-CO2 and DC-CO2 groups.

Disease-free survival was determined for the laparoscopic groups showing 82% for HW-CO2 and 72% for DC-O2 groups at 3 years, with a median follow up of 2.4 and 2.1 years, respectively; these were not significantly different. (Supplementary Fig. S9).

Surgical site infections **(**SSI) were identified in five patients or 8% in the laparoscopic cohorts; however, all were significantly (*p* = 0.02; Fisher’s exact test) within the DC-CO2 group (5/31 or 23%). Three and two patients developed SSI in the HWCO2-open and conventional-open groups, respectively (these were not significantly different). SSI are on the whole less frequent with laparoscopic surgery verses laparotomy surgery (Table 1).

## Discussion

This prospective RCT has confirmed some expectations informed by pre-clinical studies in mice and pigs^11–13,22^ as well as revealing unanticipated biology of patients undergoing surgery for CRC. The need to explore the impact of generating a pneumoperitoneum for laparoscopic surgery has been raised by others^24^ and here we addressed clinical and biological parameters in laparoscopic and open surgery cohorts.

Hypothermia during surgery and post-operatively is associated with a plethora of negative clinical factors and is evident in open and laparoscopic cases.^25^ Implicit in the design of this study was that warming CO_2_ along with humidification would impact patient core-body temperature as in mice. However, unlike mouse studies where the impact of HW-CO2 in restoring normal body temperature was almost immediate, ^11^ patients by contrast showed substantial body temperature heterogeneity where most showed a temperature drop at 15 min. It was only in the HW-CO2 laparoscopic cohort that there was a return to normothermia as a group; evident after 3 h. This is in accord with a meta-analysis of 13 laparoscopic studies where HW-CO2 insufflated patients were 0.3 °C warmer, on average, than conventional DC-CO2 insufflated patients.^7^ Here in this RCT, the average temperature difference between 210 and 315 min was 0.5 °C in favour of HW-CO2.

One enduring impact of abdominal surgery is the induction of inflammation.^26^ Inflammation is also associated with pain. Systemic CRP serves as a marker of inflammatory state and as a sentinel for sepsis.^27^ Similarly, IL6 is induced in patients undergoing major surgery^3^; the mediator of CRP production.^28^ CRP was induced in all surgery cohorts but to a greater extent (~2-fold) in open compared with laparoscopic cases. The use of HW-CO2 significantly reduced CRP levels in the first 4 days post-surgery. This elevated CRP in the DC-CO2 group may in part be compounded by SSI exclusively in this group, but this was not evident from the individual-matched data. Other cytokines that might be expected to be elevated in the context of SSI, like gamma interferon and tumour necrosis factor, declined from pre-operative levels over the following 2 days, implying that SSI were not driving the CRP differences. These observations add weight to a larger study that similarly identified doubling of SSI in DC-CO2 cases versus HW-CO2.^8^ No significant differences in severe pain measures were evident, although modest impacts of HW-CO2 have been reported^5,29^.

The statistical parameters used to power this study relied upon published animal studies^11^; either mice or pigs,^13^ but in all cases the animals were young and healthy. No evidence of any perturbations to the peritoneum was observed in these animals. It was thus a surprise to consistently find peritoneal mesothelial damage across all four groups of patients at 22% (19/85). Importantly, patients had not received neoadjuvant chemo- and/or radiotherapy for their CRC prior to their surgery.

A limitation of this part of the study is that the pre-existing damage data were based on samples taken at the beginning of surgery but relied upon the generation of pneumoperitoneum, insertion of trocars and instruments and finally taking of the biopsy from the peritoneal wall. In robotic cases this was longer due to time needed for robot docking. Therefore, some minutes (2–10 min maximum) of insufflation cannot be dismissed as being relevant to damage induction; but this explanation cannot be used to explain the same degree of damage in the open cases. It was reasonable to be mindful of instrument-generated damage being responsible; however, this issue has been deliberately explored in animal studies where the nature of ultrastructural damage generated by forceps-induced damage is demonstrably different to that measured here.^11,12,22^

Many patients in this study had co-morbidities expected of this cohort, including type-2 diabetes, GORD and/or hypertension; these were distributed evenly across cohorts and no association with pre-existing peritoneal damage and these common co-morbidities was evident. Nor was there an association with elevated IL6 or CRP and pre-existing peritoneal cellular damage. The basis for this damage remains unresolved but warrants further investigation.

LOS impacts patients and hospitals whereby there is a pressing need to reduce its duration.^30^ As anticipated, patients undergoing open surgery in general had a LOS double that of laparoscopic cases; 22 compared with 10.5 days. The small number of open cases and potential patient selection needs to be considered when understanding this difference. Colectomy cases stayed of average less than half the duration of proctectomy cases; 6 verses 13.1 days. There were no apparent differences observed when comparing robot-assisted and conventional laparoscopic approach. These observations are in accord with reviews by others^31–34^ but definitive evidence of differences in LOS from phase 3 clinical trial data to separate open, laparoscopic +/− robot assistance are currently unavailable. However, an informative recent phase 3 RCT shows that lowering insufflation pressure is associated with a reduced LOS.^4^ The positive benefit of lowering insufflation CO_2_ pressure on inflammatory markers aligns with other approaches such as HW-CO2 found in this RCT.^1,2^

When colon and rectal cancer laparoscopic groups were assessed independently a statistically significant benefit of employing HW-CO2 over DC-CO2 was evident in regarding LOS. Peritoneal damage subsequent to the initiation of surgery increased significantly more in the DC-CO2 laparoscopic group but, perhaps more interesting, was that a number of cases in the HW-CO2 group sustained levels of damage below the 50% mid-point, while there were none in the DC-CO2 group. These observations are consistent with the protective effect of HW-CO2 observed in animal studies and the reason(s) for this difference in the patient samples warrants investigation.

This study has several limitations, some anticipated and others not. The impact of informed consent describing the basis for the RCT led to patients asking for HW-CO2, precluding their randomization. Furthermore, surgical practice has progressively moved to the use of laparoscopic and robot-assisted operations, making recruitment to laparotomy increasingly difficult. The interim analysis alerted the study to the unexpected peritoneal mesothelial cell damage before initiation of surgery, reducing the number of cases with “pristine” peritoneum analogous to that observed in pre-clinical studies including pigs.^13^ The patient numbers are small and by several metrics there was substantive patient heterogeneity meaning that be a larger study would have been preferable. Longer-term clinical parameters in respect to local and distant CRC recurrence will need to mature and be reported at a later time.

## Conclusions

Collectively, this study has found that the use of HW-CO2 insufflation leads to restoration of core-body temperature as expected, but this took longer than anticipated. LOS was reduced in patients if assessed based upon surgery for tumours of the colon or the rectum. HW-CO2 was associated with less inflammation and peritoneal tissue damage. Finally, the conceptual implication of reducing peritoneal damage on the propensity for pre-existing peritoneal tumour cells to imbed in the peritoneal wall has not been resolved by this study at this point, but the marked parallels in peritoneal damage across species keeps this concern in play.

## Supplementary Information

Below is the link to the electronic supplementary material.

Supplementary file1 (DOCX 1429 kb)
