# Supplementary material for: Effect of Surgical Humidification on Inflammation and Peritoneal Trauma in Colorectal Cancer Surgery: A Randomized Controlled Trial
Source: Ann Surg Oncol. 2022 Jul 6;29(12):7911–20. doi: 10.1245/s10434-022-12057-3 (PMC9261208; doi:10.1245/s10434-022-12057-3)
Supplement: Supplementary file 1 — Supplementary file1 (DOCX 1429 kb) [file 10434_2022_12057_MOESM1_ESM.docx]

**Supplementary Fig. S1.** CONSORT flow chart describing laparotomy arms

**Supplementary Fig. S2.** Peritoneal damage to mesothelial cells (**a**) and microvilli damage (**b**) in patients undergoing laparoscopic surgery subject to HW-CO2 or DC-CO2

**Supplementary Fig. S3.** Pain and analgesia management of patients

**Supplementary Fig. S4.** IL6 levels in patients following surgery

**Supplementary Fig. S5.** IL8 and MCP-1 levels in patients following surgery

**Supplementary Fig. S6.** Cytokine levels in patients following laparoscopic surgery

**Supplementary Fig. S7.** Individual patient core body temperatures during laparoscopic insufflation

**Supplementary Fig. S8.** Patient core body temperatures in open surgery cases. (**a**) Individual patient core temperatures during open surgery are shown. Only one patient failed to exceed hypothermia above 35 ^o^C throughout the operation and was excluded from the analysis in panels (**c**) and (**d**). (**b**) HW-CO2 group started on average 0.4 ^o^C colder than the conventional group (**c**). (**d**) Their relative recovery appeared to be more rapid than that of the conventional group as measured by temperature change (ΔTemp)

**Supplementary Fig. S9.** Progression-free survival in laparoscopic cases
